# Supplementary material for: Histone H3 binding to the PHD1 domain of histone demethylase KDM5A enables active site remodeling
Source: Nat Commun. 2019 Jan 9;10:94. doi: 10.1038/s41467-018-07829-z (PMC6327041; doi:10.1038/s41467-018-07829-z)
Supplement: Supplementary file 1 — Supplementary Information [file 41467_2018_7829_MOESM1_ESM.pdf]

Supplementary information

Histone H3 binding to the PHD1 domain of histone demethylase KDM5A enables active site remodeling

Longbotham *et al*.

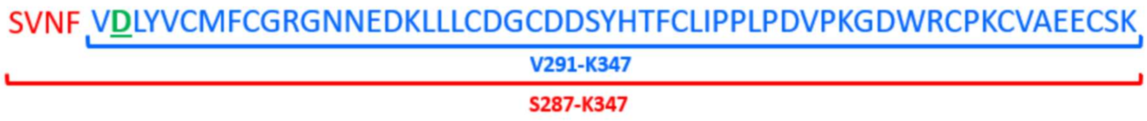

Supplementary Figure 1.

Comparison of PHD1 constructs used in this study.

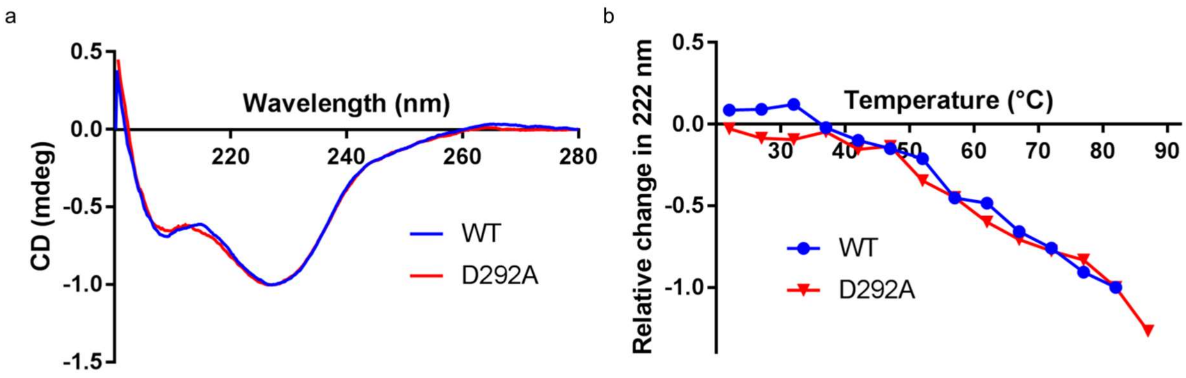

Supplementary Figure 2.

Comparison of WT PHD1 and D292A PHD1 by circular dichroism. a) CD spectra of WT $PHD1_{287\text{-}347}$ and D292A $PHD1_{287\text{-}347}$ taken at 25 °C. b) Change in CD at 222 nm after WT $PHD1_{287\text{-}347}$ and D292A $PHD1_{287\text{-}347}$ are denatured by increasing temperature. Errors (n≥3) represent s.e.m.

a

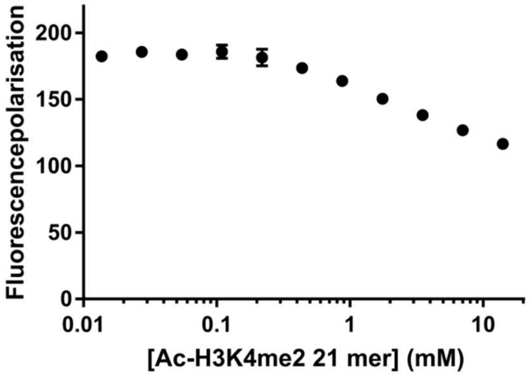

b

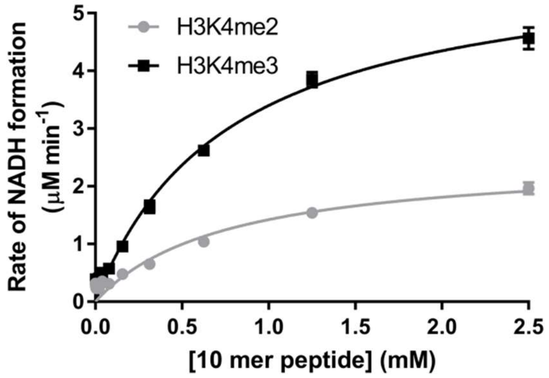

c

	H3K4me2 10mer	H3K4me3 10mer	H3K4me3 18mer	H3K4me3 21mer	H3K4me3 25mer
k_{cat} (min^{-1})	2.05 ± 0.35	4.97 ± 0.44	2.1 ± 0.04	1.8 ± 0.07	1.05 ± 0.04
K_m (μM)	750 ± 310	770 ± 170	26.5 ± 2.5	9.5 ± 1.5	3.1 ± 0.5
k_{cat}/K_m (mM^{-1} min^{-1})	2.72 ± 1.59	6.42 ± 1.94	79.3 ± 9.0	189 ± 37	339 ± 66

Supplementary Figure 3.

Binding and kinetics of KDM5A-mediated demethylation of various H3K4 peptides. a) Analysis of Ac-H3K4me2-FAM binding to $PHD1_{S287-K347}$ domain by fluorescence polarization. b) Michaelis-Menten analysis of KDM5A-catalyzed demethylation of di- and trimethylated H3K4 10mer peptides. Activity was measured by the formaldehyde release assay. c) Kinetics parameters for KDM5A-mediated demethylation of H3K4 methylated peptides of different lengths as measured by the formaldehyde release assay. Errors (n≥3) represent s.e.m.

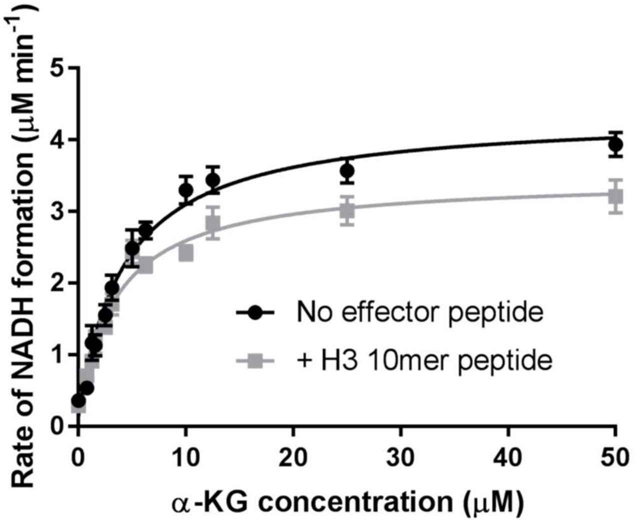

	No effector peptide	+ H3 10mer peptide
k_{cat} (min^{-1})	4.36 ± 0.17	3.47 ± 0.14
K_m (μM)	4.27 ± 0.71	3.64 ± 0.66
k_{cat}/K_m (mM^{-1} min^{-1})	1.02 ± 0.21	0.95 ± 0.21

Supplementary Figure 4.

Michaelis-Menten kinetics of α-KG turnover by KDM5A. The activity of KDM5A was determined with varying concentrations of α-KG in the presence and absence of 38 μM H3 10mer peptide. 1μM of KDM5A and 1 mM of Ac-H3K4me3 21mer peptide substrate were used and activity was measured by the formaldehyde release assay. Errors (n≥3) represent s.e.m.

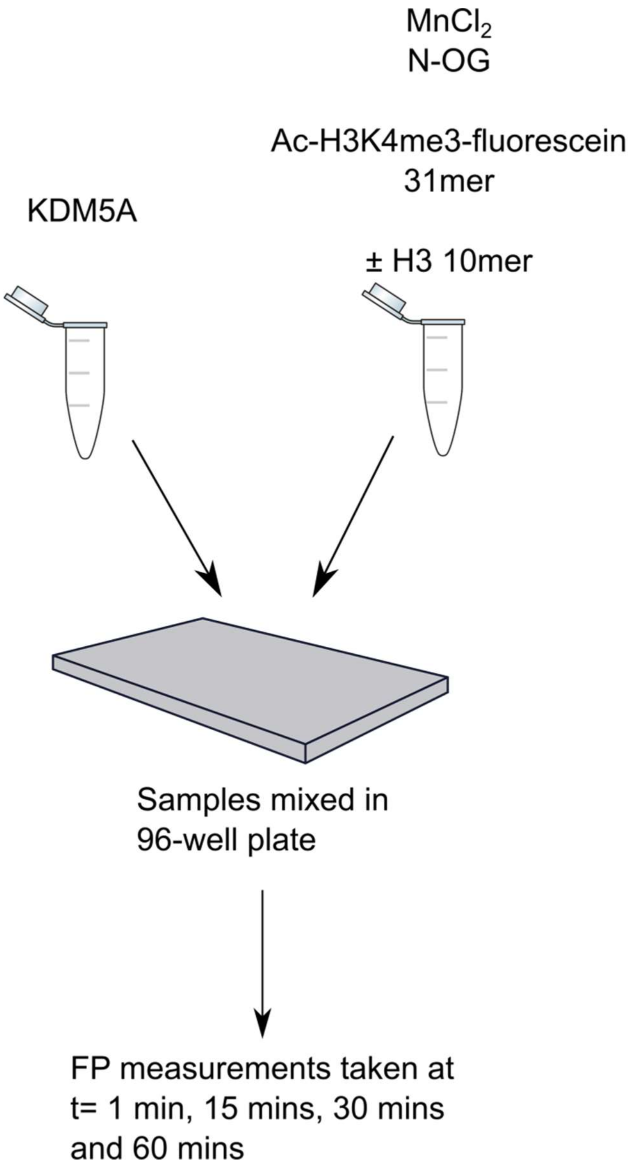

Supplementary Figure 5.

Schematic of the direct binding assay of substrate to KDM5A. Varying concentrations of KDM5A were mixed with a solution of $MnCl_2$, N-OG and Ac-H3K4me3-fluorescin 31mer peptide in a 96 well plate. This was done either in the presence and absence of the H3 10mer peptide. The fluorescence polarization of these samples was immediately measured using a plate reader at 1, 15, 30, 45 and 60 mins.

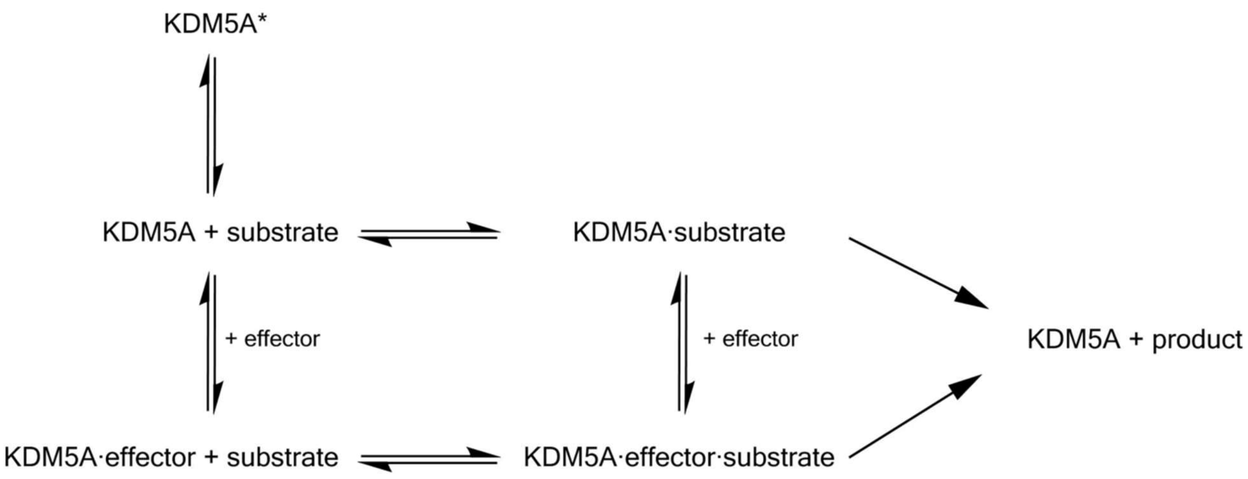

Supplementary Figure 6.

Proposed mechanism for improved substrate binding in KDM5A. We suggest that KDM5A alternates between an "open" (KDM5A) and "closed" (KDM5A*) state, where the closed state is not optimal for substrate binding. The slow equilibrium between the two states, as suggested by our data, is potentially responsible for the decrease in substrate binding we observe in Figure 3a. Upon effector peptide binding to the PHD1 domain this equilibrium is shifted to the more "open" state where it is able to stably bind substrate (**Figure 3b**). According to the data we have presented (**Figure 3**) binding of the substrate appears fast as a binding curve is observed after 1 min for both "apo" and effector peptide bound conformations.

a

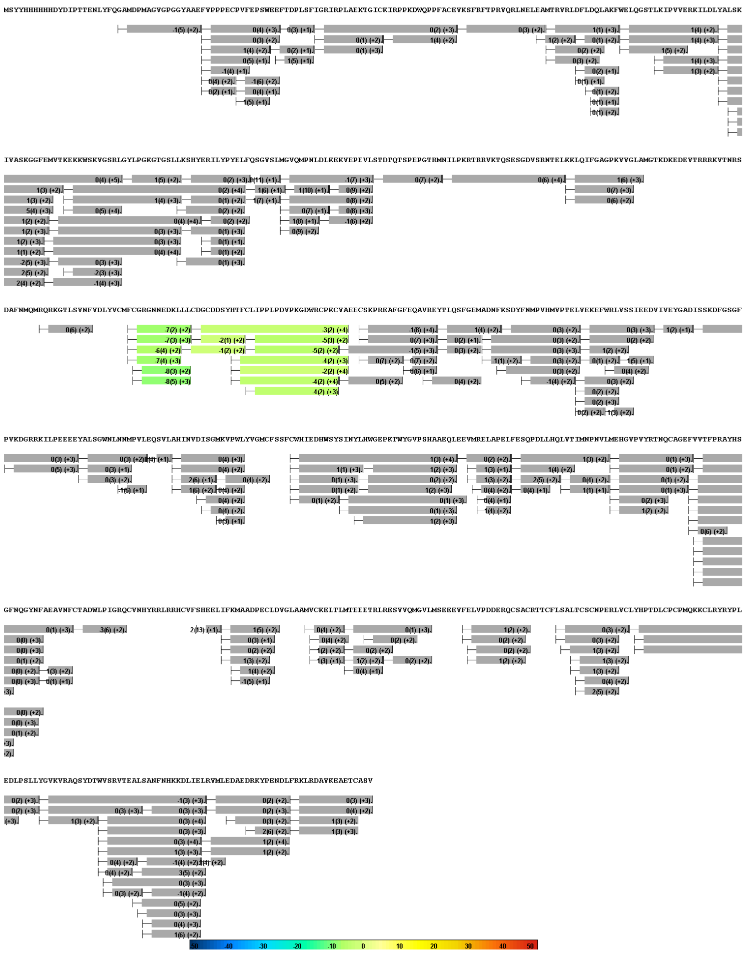

b

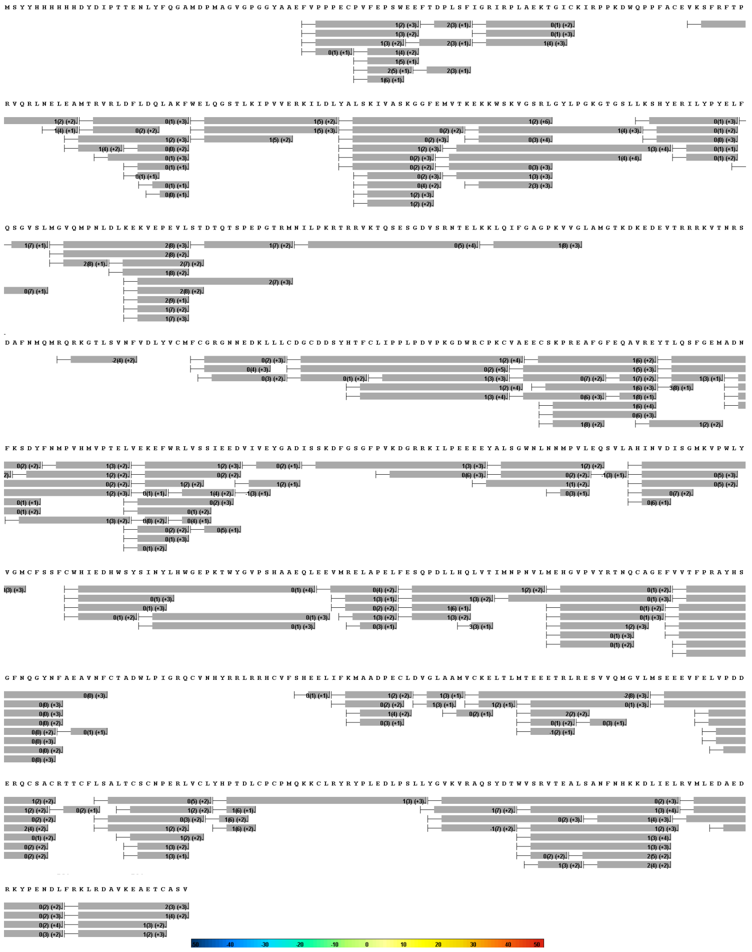

c

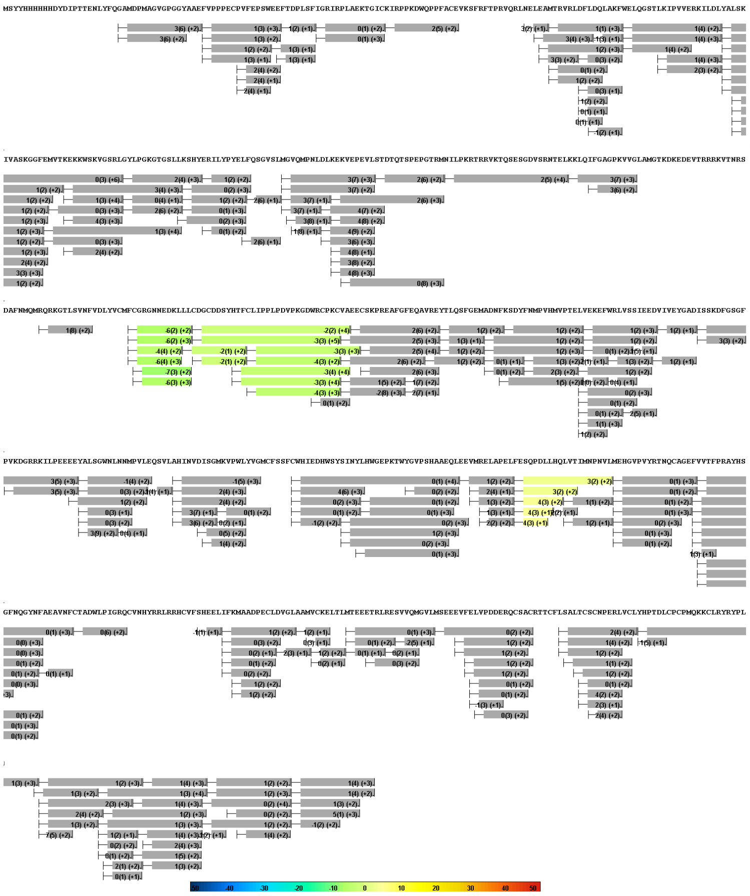

Supplementary Figure 7.

Percentage difference in HDX of KDM5A. a) KDM5A·Fe(II)·N-OG (apo KDM5A) compared with KDM5A·Fe(II)·N-OG·H3 10mer b) KDM5A·Fe(II)·N-OG (apo KDM5A) compared with KDM5A·Fe(II)·N-OG·Ac-H3K4me3 21mer. c) KDM5A·Fe(II)·N-OG (apo KDM5A) compared with KDM5A·Fe(II)·N-OG·H3 10mer· Ac-H3K4me3 21mer. Errors displayed are described in the methods and results.

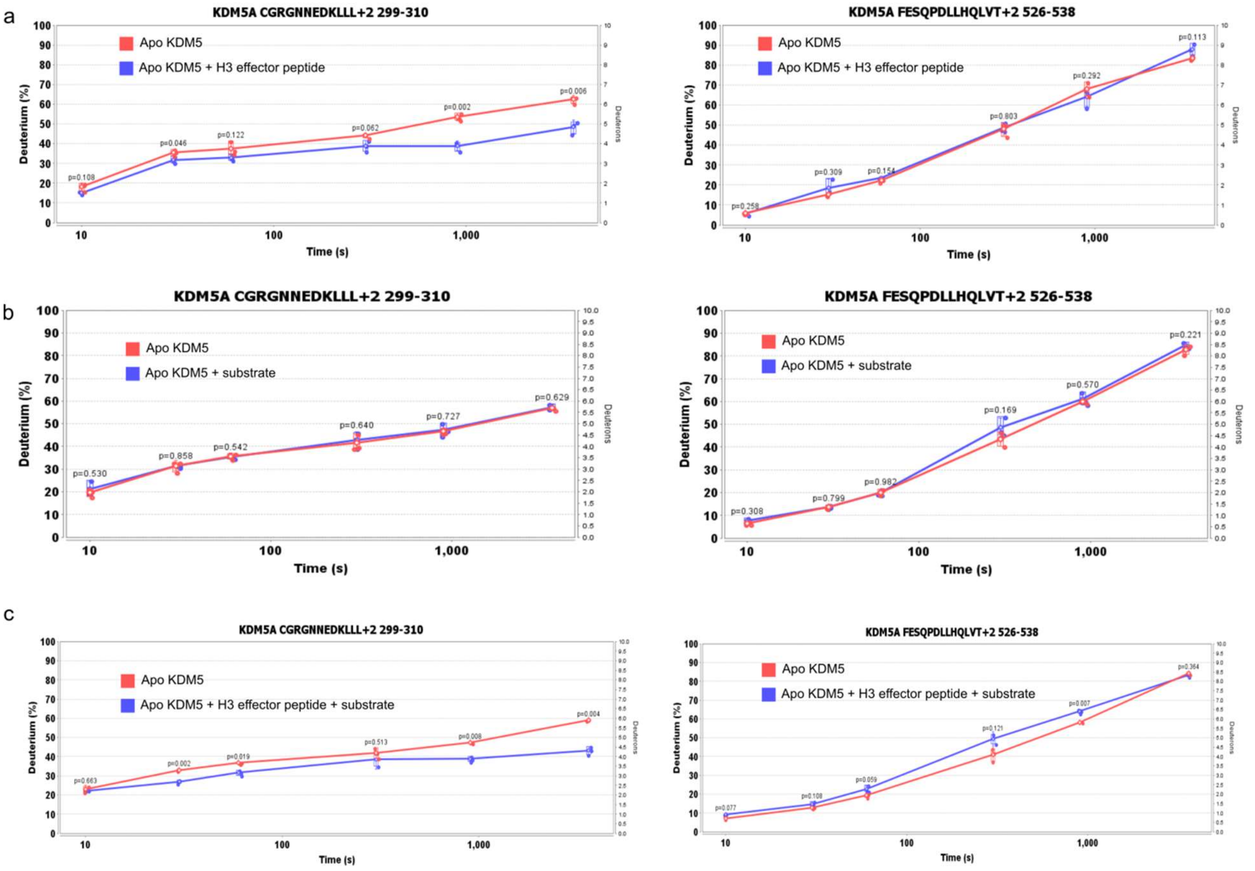

Supplementary Figure 8.

Deuterium uptake in regions of KDM5A over time in the presence of different peptides. a) KDM5A·Fe(II)·N-OG (apo KDM5A) compared with KDM5A·Fe(II)·N-OG and 100 µM H3 10mer. b) KDM5A·Fe(II)·N-OG (apo KDM5A) compared with KDM5A·Fe(II)·N-OG with 500 µM Ac-H3K4me3 21mer. c) KDM5A·Fe(II)·N-OG (apo KDM5A) compared with KDM5A·Fe(II)·N-OG with 100 µM H3 10mer and 500 µM Ac-H3K4me3 21mer. Errors displayed are described in the methods and results.

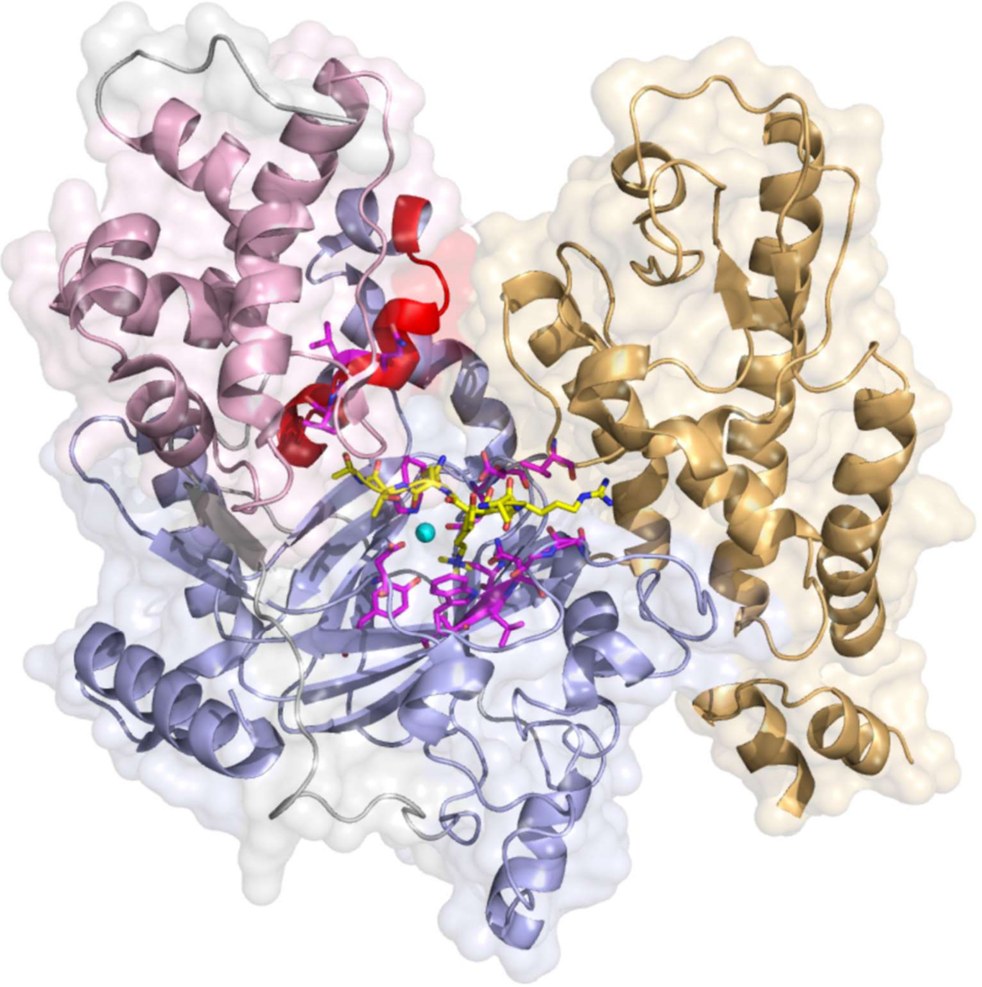

Supplementary Figure 9.

Structural analysis of a potential peptide binding site in KDM5A

The structure of KDM5A (PDB ID: 5CEH) overlaid with structure of homologue JMJ14 (PDB ID: 5YKO), which is hidden for clarity. The H3K4me3 ligand bound in the JMJ14 structure is shown in yellow. Residues of KDM5A that could potentially interact with the H3K4me3 7mer ligand are shown in magenta. Many of these residues are conserved between KDM5A and JMJ14. These

are Asp412, Ser464, Gly465, Trp470, His 483, Glu485, Asp486, Ser491, Val537, Ala583, Val584, Asn585, Val610. The Ni atom is show in cyan.

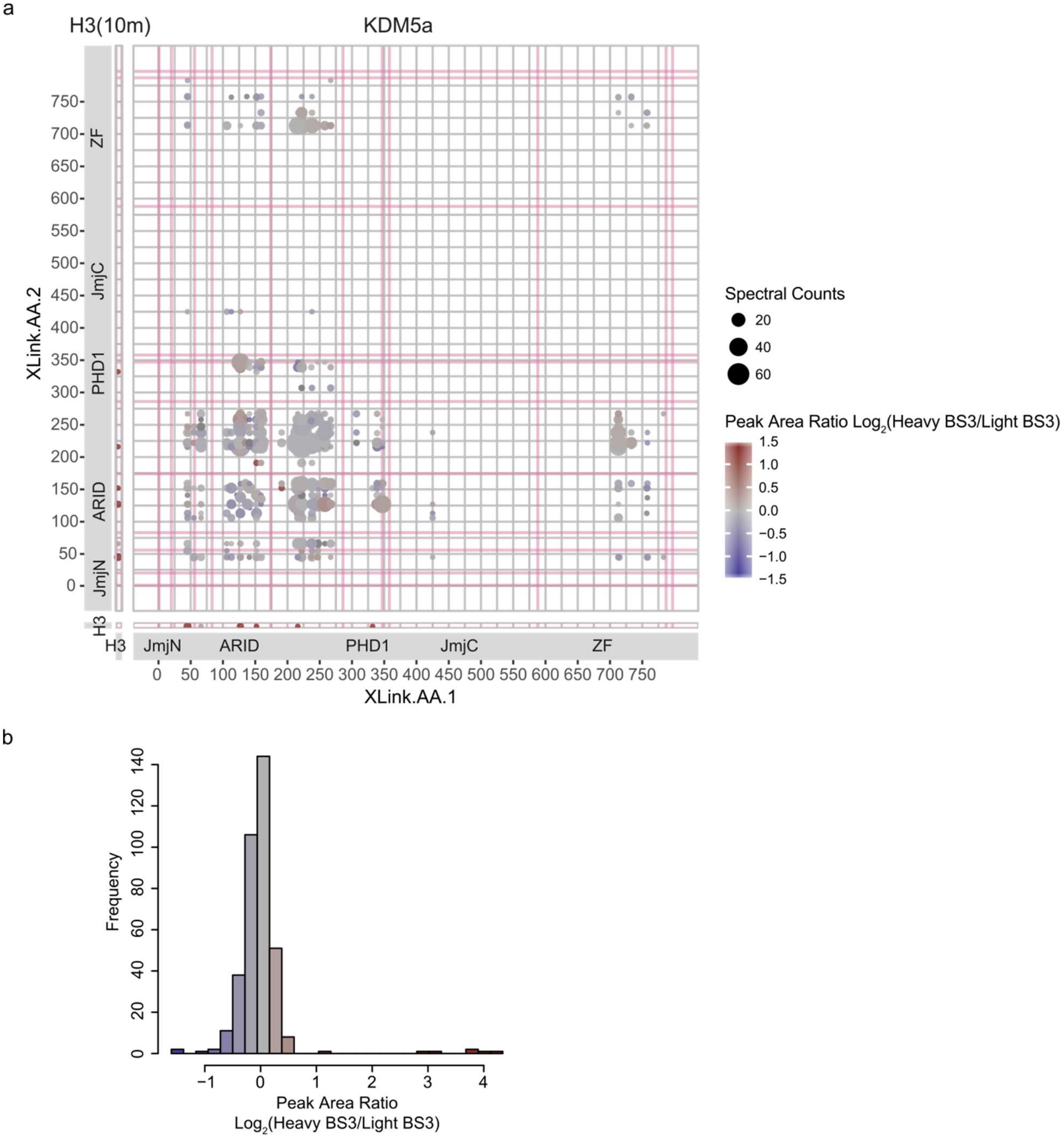

Supplementary Figure 10.

Crosslinking Mass Spectrometry to monitor the dynamics of KDM5A. KDM5A in the presence and absence of H3 10mer effector peptide was crosslinked with lysine directed reagents, $^{2}H_{12}$-BS3 and $^{1}H_{12}$-BS3, respectively. The reaction mixtures were mixed prior to tryptic

digestion and crosslinked residue pairs were identified by mass spectrometry. The ratios of heavy to light crosslinks were determined from the extracted peak areas in the precursor scan. a) Each point represents a crosslink between a pair of amino acid residues whose sequence position within the H3-10mer and KMD5A are given by the x- and y-axis. KDM5A domain boundaries are demarcated by pink lines. The area of each point is proportional to the number of product ion spectra identifying a crosslink, while the color scale indicates whether a crosslink was enriched in presence of effector peptide (red) of absence (blue). b) The overall distribution of crosslinked heavy to light peak areas, plotted as log2 ratios. Aside from crosslinks between the effector peptide and KDM5A, the majority of intra-protein KDM5A crosslinks exhibit only modest (less than 2-fold) changes in peak area ratio.
